# Electronic and structural properties of Möbius boron-nitride and carbon nanobelts

**DOI:** 10.1186/s11671-024-03967-0

**Published:** 2024-04-08

**Authors:** C. Aguiar, N. Dattani, I. Camps

**Affiliations:** 1 Laboratório de Modelagem Computacional - LaModel, Instituto de Ciências Exatas - ICEx, Universidade Federal de Alfenas - UNIFAL-MG, Alfenas, Minas Gerais Brazil; 2 HPQC College, Waterloo, Canada; 3 HPQC Labs, Waterloo, Canada

**Keywords:** Nanotechnology, Nanobelts, Boron nitride, Carbon

## Abstract

For the development of nanofilters and nanosensors, we wish to know the impact of size on their geometric, electronic, and thermal stabilities. Using the semiempirical tight binding method as implemented in the xTB program, we characterized Möbius boron-nitride and carbon-based nanobelts with different sizes and compared them to each other and to normal nanobelts. The calculated properties include the infrared spectra, the highest occupied molecular orbital (HOMO), the lowest unoccupied molecular orbital (LUMO), the energy gap, the chemical potential, and the molecular hardness. The agreement between the peak positions from theoretical infrared spectra compared with experimental ones for all systems validates the methodology that we used. Our findings show that for the boron-nitride-based nanobelts, the calculated properties have an opposite monotonic relationship with the size of the systems, whereas for the carbon-based nanobelts, the properties show the same monotonic relationship for both types of nanobelts. Also, the torsion presented on the Möbius nanobelts, in the case of boron-nitride, induced an inhomogeneous surface distribution for the HOMO orbitals. High-temperature molecular dynamics also allowed us to contrast carbon-based systems with boron-nitride systems at various temperatures. In all cases, the properties vary with the increase in size of the nanobelts, indicating that it is possible to choose the desired values by changing the size and type of the systems. This work has many implications for future studies, for example our results show that carbon-based nanobelts did not break as we increased the temperature, whereas boron-nitride nanobelts had a rupture temperature that varied with their size; this is a meaningful result that can be tested when the use of more accurate simulation methods become practical for such systems in the future.

**Supplementary Information:**

The online version contains supplementary material available at 10.1186/s11671-024-03967-0.

## Introduction

Carbon atoms can present three different forms of hybridization (*sp*, $sp^2$, and $sp^3$), and the way in which they can combine with other elements is the basis of countless research studies [1, 2]. The combination of this element on a nanometric scale gave rise to structures called nanocarbons [3]. They are characterized by showing different geometric and dimensional configurations, such as fullerenes, carbon nanotubes, graphene nanoribbons, graphene oxides, and nanodiamonds [1, 4]. Such structures have attracted interest for promising applications in nanobiomedicine [5, 6] and optoelectronics [7, 8]. Furthermore, carbon-based nanomaterials are highly biocompatible with living organisms and the environment [9].

According to their size and/or different topologies, these nanomaterials cause different reactivity when in contact with other substances [2, 10], favored or not, according to the geometry of the nanomaterial, which varies according to the increase in the pyramidalization angle and misalignment of the $\pi$ orbitals between the C atoms [2]. Thus, different syntheses and functionalization have been carried out to obtain different nanostructures to achieve greater compatibility with other materials, as we seek to improve and expand nanotechnological applications [5, 10–12].

Povie et al. made a breakthrough with the synthesis of carbon nanobelts (CNBs), whose simple structure in the form of a ring or belt generates two faces, one internal and one external, not convertible to each other [13]. Carbon nanobelts represent segments of single-walled carbon nanotubes containing a benzene ring cycle with *p* orbitals aligned in a plane [14]. Such behavior allows them to be classified as armchair, zigzag, or chiral nanoribbons according to the chirality index [14, 15]. In addition to the ring shape, carbon nanobelts are interesting structures due to their synthetic challenges and differentiated properties [16, 17]. Its formulation covers concepts of conjugation, aromaticity, and strain, as well as important information on chirality and bottom-up synthesis of C nanotubes, which continues to be a challenge and has caused limitations in its applications [18]. For the synthesis of nanobelts, three steps are typically considered: the first consists of macrocyclization from carbon sources; the second is the formation of the belt in order to create the double-stranded structure; and finally, the induction step for the bending of the $sp^2$ hybridized carbon skeleton into a cylindrical topology [13, 19].

In addition, CNBs can be “twisted” to acquire a Möbius topology. The first organic systems synthesized with Möbius topologies were Möbius annulenes [20, 21] and thereafter, Möbius molecules of various types have been created and investigated since then [18, 22–25].

Möbius carbon nanobelts (MCNBs), manifest different properties and molecular movements when compared to common nanobelts [13]. Density functional theory (DFT) calculations show that MCNBs have a higher strain energy than ordinary CNBs of the same size [18]. However, producing torsion in ordinary CNBs can be difficult to control, as the strain energy is the major obstacle in the synthesis of MCNBs [13]. For this, saturated ligands (-CH_2_O-) or chalcogen atom ligands (-S-) are used to reduce and control the stress caused by the Möbius shape [26, 27]. Nonetheless, calculations of strain energies showed that MCNBs are synthetically accessible and that strain decreases with increasing MCNB size [21]. Data from nuclear magnetic resonance spectroscopy and theoretical calculations show that the torsion structure of the Möbius band moves rapidly in solution [18]. Furthermore, chirality arising from the Möbius structure has been demonstrated experimentally using chiral separation by high-performance liquid chromatography (HPLC) and circular dichroism (DC) spectroscopy [18]. Besides, spectroscopy data on excitation at 380 nm showed blue–green fluorescence beyond 10% quantum yield [18].

Carbon atoms in carbon-based nanostructures can be replaced by boron and nitrogen atoms, resulting in boron-nitride (BN) isoform structures, for example, carbon nanotubes (CNTs) can be replaced by boron-nitride nanotubes (BNNTs) [28]. With an analogous structure to CNTs, BNNTs have similar mechanical properties [28, 29] and they are electrically insulating, with a forbidden band between 5.0$-$6.0 eV, independent of chirality [28, 30]. In addition, the thermal properties are improved due to boron-nitride having high atmospheric stability, especially at high temperatures [31]. This makes BNNTs excellent candidates for applications in electronics [32–36], sensors [37], hydrogen storage [38], medicine [39, 40], and also water purification [41–46], among others.

Boron-nitride nanobelts (BNNBs), an inorganic analog of cyclophenazine, were synthesized in 2017 [47, 48]. Aromatic BNNBs have radially oriented *p* orbitals with photoluminescent properties and are excellent UV absorbers, suggesting that such systems can be used as UV detectors [13, 48, 49]. In addition, boron-nitride nanobelts have high chemical stability, thermal stability with positive vibrational frequencies, and insulating character with an estimated gap of around 5 eV [48, 50]. Several, *in vitro* and *in vivo* studies were conducted with boron-nitride nanostructures of different shapes and sizes in order to determine their cytotoxicity. The studies concluded that the pristine forms of boron-nitride structures without coatings turn out to be bio-compatible *in vitro* and *in vivo*. This makes BN materials attractive for bio-applications [51–54].

In this work, for the first time, the structural and electronic properties as well as thermal stabilities of Möbius and ordinary carbon and boron-nitride nanobelts are investigated as a function of their size for up to five different sizes. Knowing how the size affects the nanostructure properties [55–59] allows us to control such properties by modifying the growth processes.

## Materials and methods

In this work, two types of CN nanobelts and two types of BN nanobelts were used: ordinary and Möbius (twisted) nanobelts for each case. Structures were generated starting with a cell with 2 units of (10,0) nanosheets repeated *n* times (10, 15, 20, 25, and 30) in the z-direction and then wrapped $360^\circ$. After that, the periodicity was removed, and the atoms at the borders were passivated with hydrogen. In the case of Möbius nanobelts, after repetition, the nanobelts were twisted $180^\circ$ and then wrapped. All the structures were generated using the Virtual NanoLab Atomistix ToolKit software [60]. The nomenclature to identify the systems is described as follows: BNNB*n* (CNB*n*) for boron-nitride (carbon) nanobelts and MBNNB*n* (MCNB*n*) for Möbius boron-nitride (carbon) nanobelts. In all cases, *n* indicates the number of repetitions.

Using the semiempirical tight binding method as implemented in the xTB (eXtended Tight Binding) program [61], the electronic and structural parameters were calculated. The calculations were done using the GFN2-xTB method (GFN is an acronym for geometries, frequencies, and noncovalent interactions, and the number 2 corresponds to the version of the method). GFN2-xTB is an accurate self-consistent method that includes multipole electrostatics and density-dependent dispersion contributions [62], with an extreme optimization level that ensures a convergence energy of $5\times 10^{-8}$ E_h_ and a gradient norm convergence of $5\times 10^{-5}$ E_h_/a_0_ (E_h_ is the Hartree unit, and a_0_ is the Bohr radius). The files with the orbital information were treated with the ORBKIT software [63] that creates the correct inputs for visualization with VMD [64].

For each optimized structure, the highest occupied molecular orbital, HOMO ($\varepsilon _H$); the lowest unoccupied molecular orbital, LUMO ($\varepsilon _L$); the energy gap ($\Delta \varepsilon$) between HOMO and LUMO orbitals ($\Delta \varepsilon = \varepsilon _H - \varepsilon _L$); the chemical potential ($\mu$); the molecular hardness ($\eta$); and the infrared spectra were determined.

Considering the approximation that ignores orbital relaxation after an electron is removed from the system (Koopmans’ s theorem [65–68] together with Janak’ s theorem [69]), it is possible to estimate the chemical potential ($\mu$), the molecular hardness ($\eta$) [70], and the electrophilicity index ($\omega$) [71] from the HOMO and LUMO energies $\epsilon _{H}$ and $\epsilon _{L}$ as follows:

1 $$\begin{aligned}{} & {} \mu \cong \frac{{{\varepsilon _L} + {\varepsilon _H}}}{2},\end{aligned}$$

2 $$\begin{aligned}{} & {} \quad \eta \cong \frac{{{\varepsilon _L} - {\varepsilon _H}}}{2},\end{aligned}$$

3 $$\begin{aligned}{} & {} \quad \omega = \frac{{{\mu ^2}}}{{2\eta }}. \end{aligned}$$

## Results and discussion

**Fig. 1:**
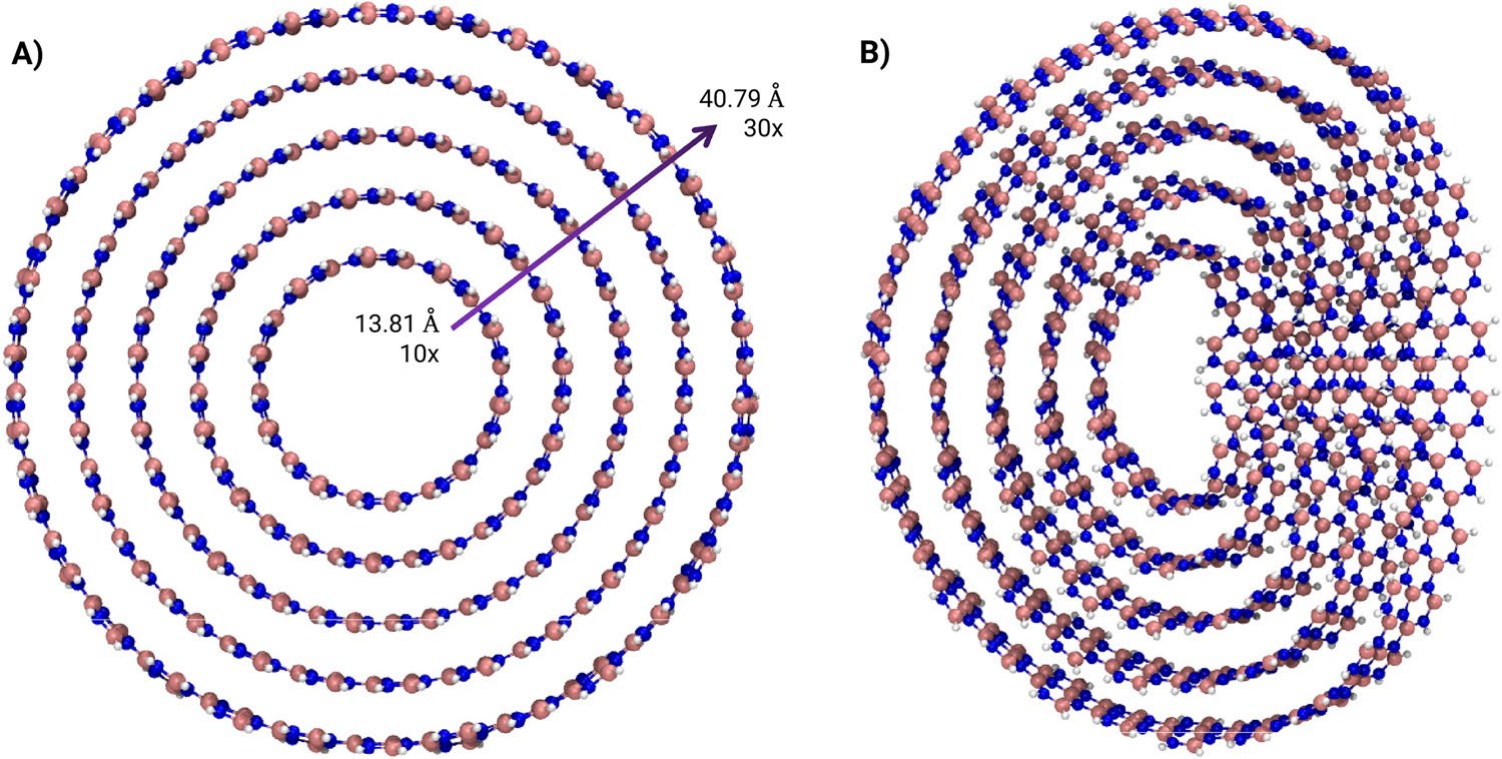
Top view of: **A** Boron-nitride nanobelts (BNNBs) with minimum/maximum diameter and repetition. **B** Möbius boron-nitride nanobelts (MBNNBs). Image rendered with VMD software [64]

**Fig. 2:**
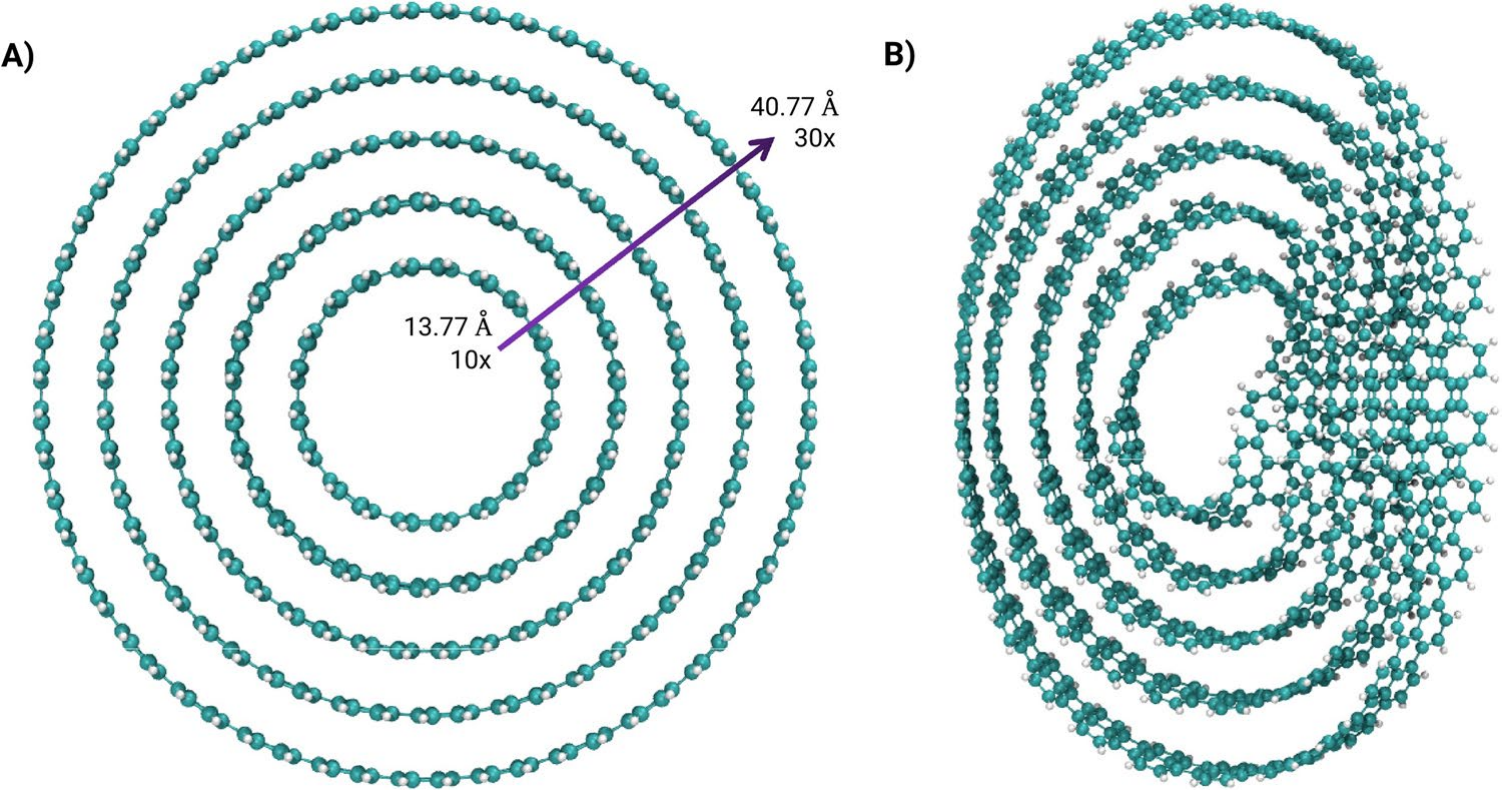
Top view of: **A** Carbon nanobelts (CNBs) with minimum/maximum diameter and repetition. **B** Möbius carbon nanobelts (MCNBs). Image rendered with VMD software [64]

Figures 1 and 2 show the top view of the optimized structures. Panel A shows the ordinary nanobelts, and Panel B shows the Möbius nanobelts. With the increase in repetitions (*n*), the diameter increases too, starting from 13.81 Å (13.77 Å) to 40.79 Å (40.77 Å) for boron-nitride (carbon) nanobelts. The minimum number of repetitions used was 10 to avoid stressed structures.

**Fig. 3:**
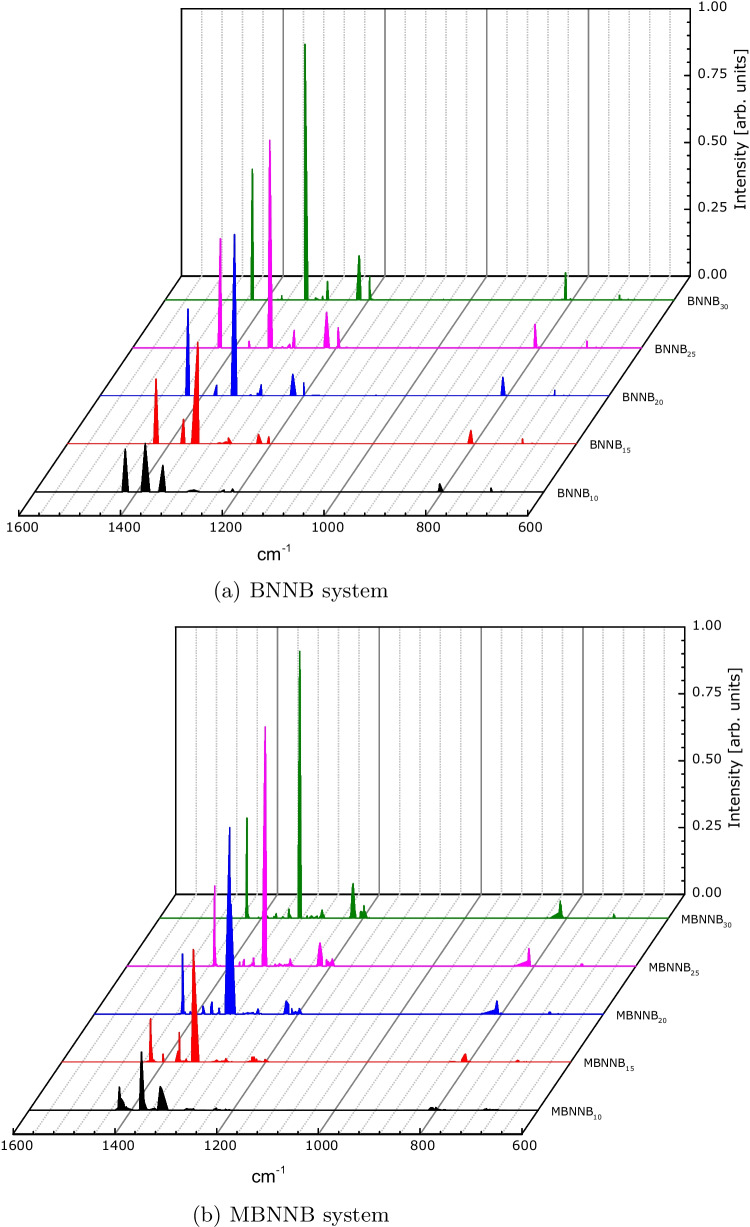
Calculated infrared spectra for boron-nitride nanobelts

**Fig. 4:**
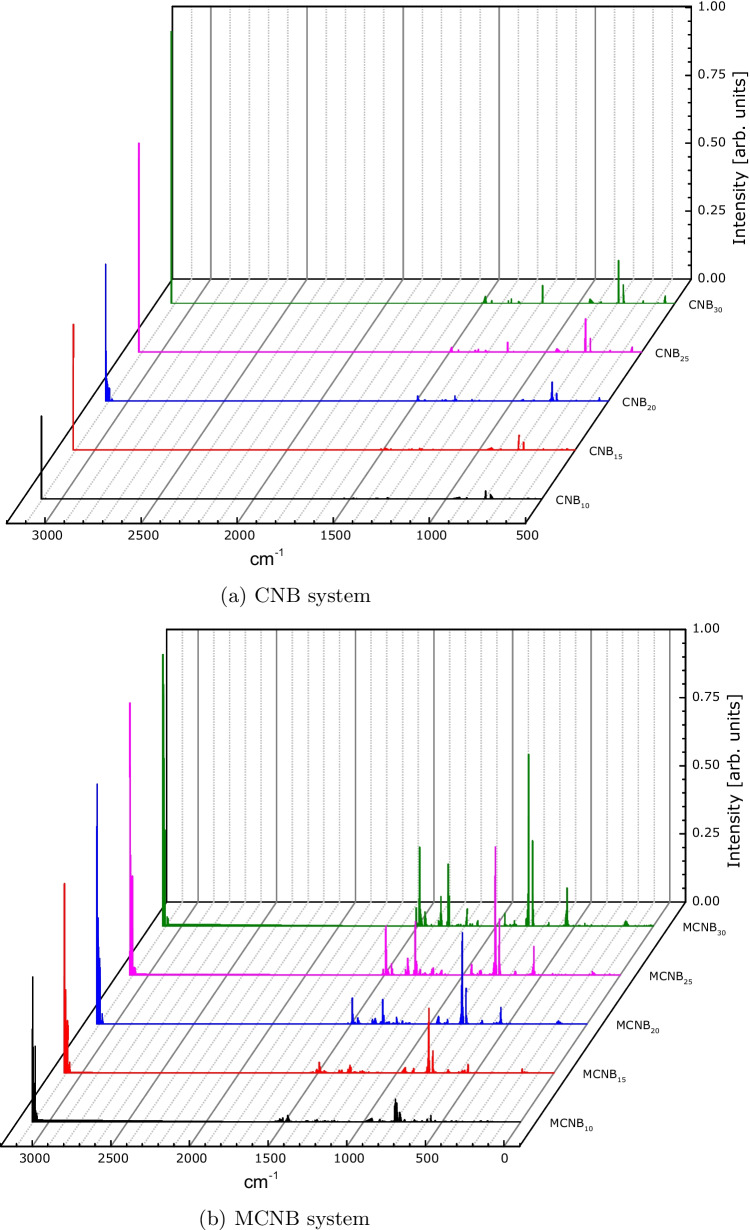
Calculated infrared spectra for carbon nanobelts

The calculated infrared spectra for each system are shown in Figs. 3 and 4. At first sight (Fig. 3), the spectra for both systems, BNNB and MBNNB, are very similar, indicating that the torsion on the Möbius nanobelts did not appreciably change the principal oscillation modes. In both cases, B-N stretching (in-plane and out-of-plane) and radial R mode (out-of-plane buckling) are observed. The oscillations around 800 cm^−1^ correspond to the out-of-plane buckling mode. All the resonances in the high-frequency regime above 1200 cm^−1^ consist of transverse optical (T) and longitudinal optical (L) phonon modes. Oscillations around 1200 cm^−1^ and 1380 cm^−1^ correspond to bond-bending or T modes, and around 1340 cm^−1^ and 1420 cm^−1^ correspond to bond-stretching or L modes [72–75].

The case for the carbon nanobelt systems, (CNBs and MCNBs), is different. For both systems, the fingerprint region ($1500-600$ cm^−1^) is visible, but the peaks have very dissimilar intensities, being greater for the MCNB structures. For carbon-based organic systems, the resonances around $900-675$ cm^−1^ were identified as out-of-plane C–H oscillations, the resonances around $1500-1400$ cm^−1^ were identified as C–C stretches (in-ring modes), and the resonances around $3100-3000$ cm^−1^ were identified as C–H bond stretches [76]. These results indicate that the MCNBs vibrate more than the CNBs.

**Fig. 5:**
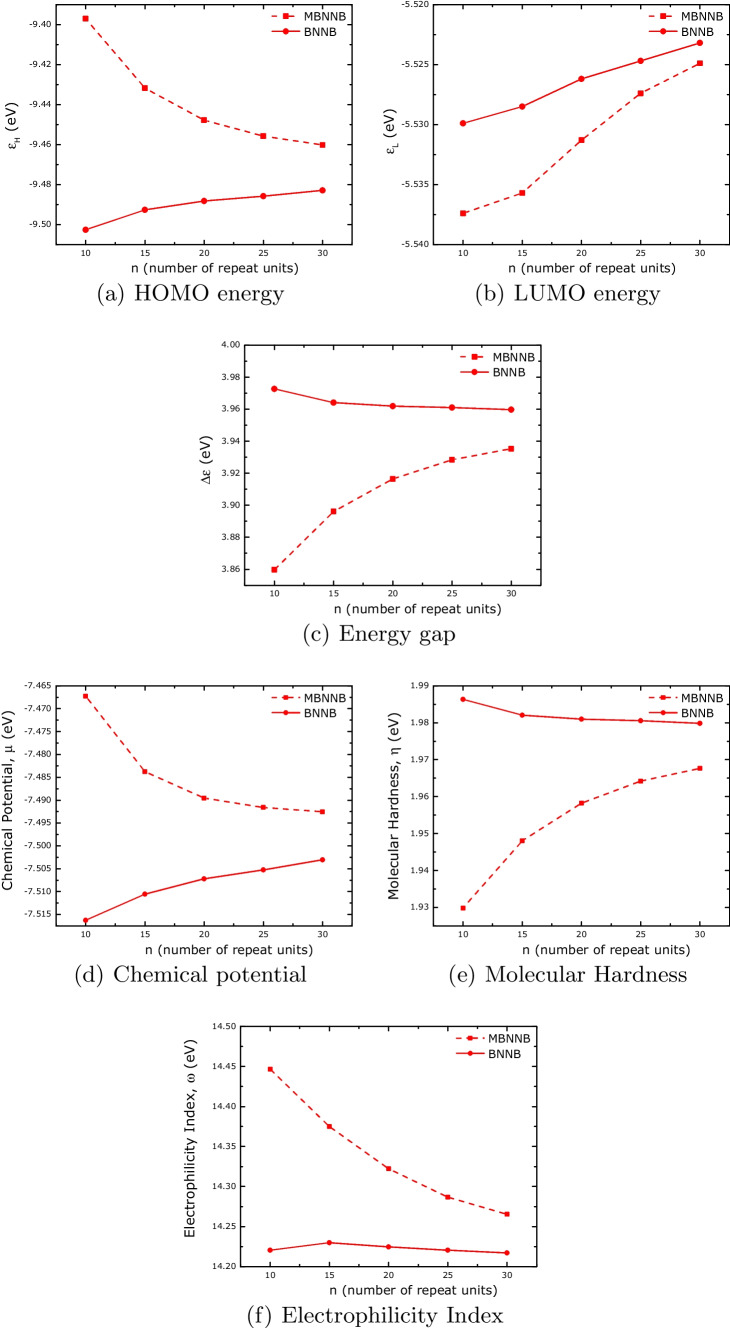
Calculated electronic properties for boron-nitride nanobelts

The electronic properties of boron-nitride and carbon-based systems are shown in Fig. 5. Figure 5a and b show the energies of the HOMO and LUMO boundary orbitals. To some extent, from the energies of these orbitals, it is possible to know how reactive the system is. The electron-donor character (electron-donor capacity) is measured by the HOMO energy, whereas the electron-acceptor character (resistance to accepting electrons) is measured by the LUMO energy. From these figures, we can see that, in the case of HOMO energy, the BNNB and MBNNB have opposite behavior. With the increase in the number of repeat units, the capacity to donate electrons decreases for the MBNNB system and increases for the BNNB. On the other hand, the behavior of LUMO energy with an increase in system size is similar, increasing the resistance to accept electrons.

**Fig. 6:**
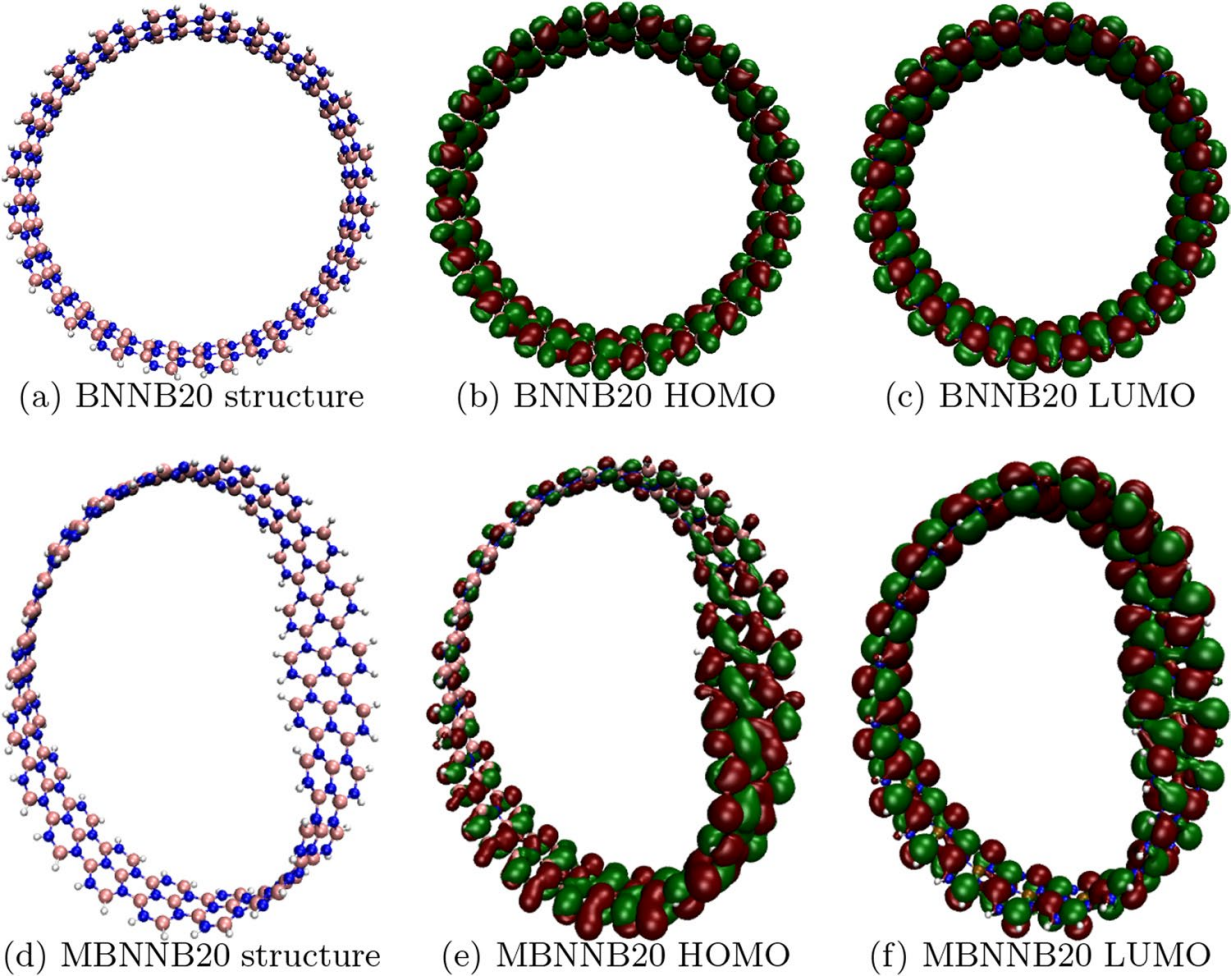
Structures, HOMO and LUMO surfaces for BNNB20 and MBNNB20 systems. Red (green) color represents negative (positive) values. Orbital surfaces rendered with isovalue equal to 0.001 and with VMD software [64]

The top view of the three-dimensional (3D) diagrams of the HOMO and LUMO surfaces for the BNNB20 and MBNNB20 are shown in Fig. 6 (the 3D diagrams for all the structures are shown in Additional file 1: Figures S1 and S2 of the Supplementary Material). The distribution of HOMO and LUMO surfaces for the BNNB is, as expected, in accordance with the symmetry of the system, being distributed homogeneously over the entire structure. In the case of the MBNNB, the torsion of the nanobelt induced a strain on the structure, which in turn modified how the orbitals were distributed. Because of the torsion, the distance between atoms alters, which is reflected in the strain produced. The distance distribution between atoms in systems with $n=20$ is illustrated in Additional file 1: Figure S5 of the Supplementary Material (the distance distribution was calculated with OVITO software [82]). In contrast to BNNB20, the distance distribution peaks for B–N, B–B, and N–N exhibited a modification in MBNNB20. We no longer have precisely defined positions, but rather a new distribution that extends to greater and smaller distances. A reduction in the superposition of wavefunctions occurs as the atoms undergo separation. Conversely, the superposition of wavefunctions increases as the distance between atoms decreases. This modification is stronger for the HOMO, where the orbital volume is redistributed into regions with smaller or greater volumes. For this iso-value, the larger volume suggests a larger probability of finding the electrons, the electron-donor regions change too, with low- or high-localized HOMO zones. On the other hand, the LUMO surface shows very little inhomogeneity. This behavior is the same for all the MBNNB, as shown in Additional file 1: figure S2 of the Supplementary Material.

The gap ($\Delta \varepsilon$), chemical potential ($\mu$), molecular hardness ($\eta$), and electrophilicity index ($\omega$) can be used to estimate the chemical reactivity and hardness of the system together with its molecular stability. Molecules with a high (or low) gap are those with high (or low) molecular stability [77].

Positive values of $\eta$ signify an energetically unfavorable redistribution of electrons within the molecule. Additionally, an increase in value corresponds to a greater degree of chemical stability in the molecule, which consequently makes electron rearrangement more difficult [78]. The molecule’ s energy reduction resulting from the maximum electron flow between its surroundings and itself can be quantified using the electrophilicity index ($\omega$) [71].

Comparing the values of $\Delta \varepsilon$, $\mu$, $\eta$, and $\omega$ for the BNNB and MBNNB systems, we can see that the values are on the same order of magnitude. The main difference is in how these properties change with the increase in the number of repeated units used to build the nanobelts. These can be used to design structures with fine control over these properties.

**Fig. 7:**
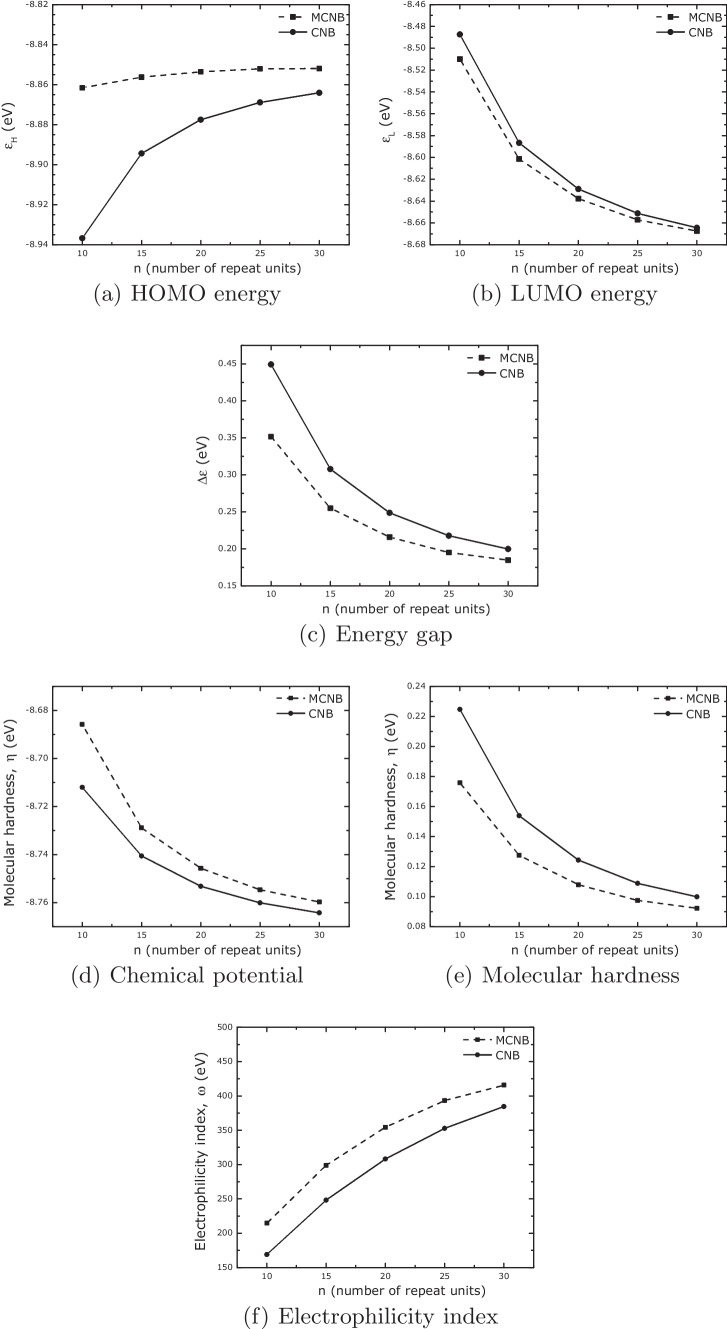
Calculated electronic properties for carbon nanobelts

Figure 7 shows the electronic properties of the carbon nanobelts. All the graphs shown a different behavior when compared to boron-nitride nanobelts. In this case, the properties for both systems, CNB and MCNB, behave with the same monotonic variation.

**Fig. 8:**
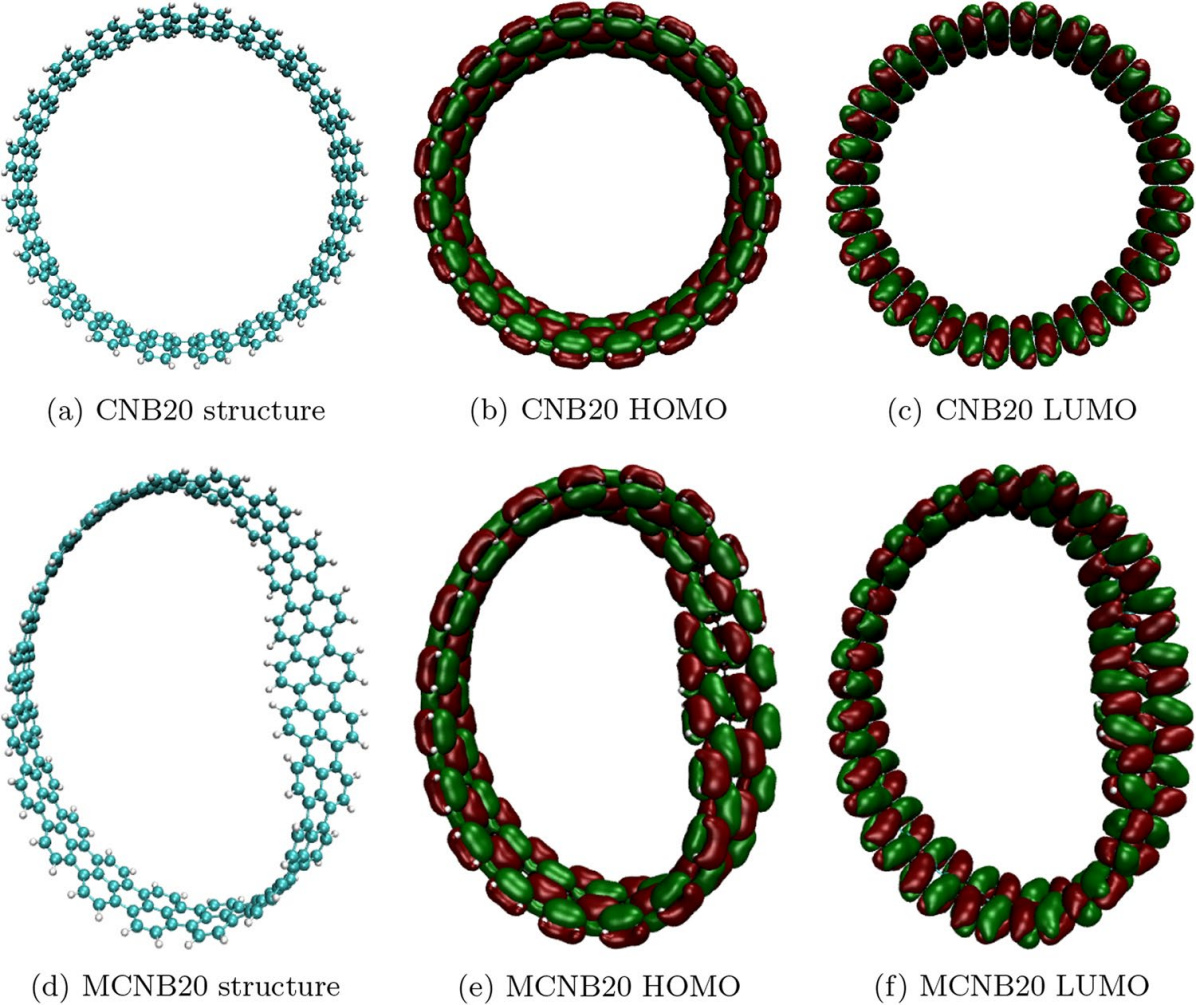
Structures, HOMO and LUMO surfaces for CNB20 and MCNB20 systems. Red (green) color represents negative (positive) values. Orbital surfaces rendered with isovalue equal to 0.001 and with VMD software [64]

The 3D top view diagrams of the HOMO and LUMO surfaces for the CNB20 and MCNB20 are shown in Fig. 8 (the 3D diagrams for all the structures are shown in Additional file 1: Figures S3 and S4 of the Supplementary Material). Because the torsion has a negligible effect on the atomic distribution of carbon nanobelts (refer to Additional file 1: Figure S5 of the Supplementary Material), neither system’s orbital surfaces were significantly altered by the torsion on the nanobelt.

The values of $\Delta \varepsilon$, $\mu$, $\eta$, and $\omega$ for CNB and MCNB systems are similar among them, with small variations with the increase in the number of repeat units used to build the nanobelts. As the properties also vary with the increase of repeat units, the change in *n* can tailor the electronic properties of the carbon nanobelts.

The inhomogeneous distribution of the HOMO surfaces can be correlated with the molecular hardness, $\eta$. As $\eta$ is related to how energetically unfavorable it is to redistribute the electrons in a molecule, higher values imply a harder rearrangement of them. Comparing the values of $\eta$ for the MBNNB (Fig. 5e) and MCNB (Fig. 7e) systems, we can see that $\eta$ is almost eight times bigger for boron-nitride nanobelts than for carbon-based nanobelts. A lower value of $\eta$ implies that the electrons can redistribute easily through the whole structure, resulting in a more homogeneous HOMO surface.

**Fig. 9:**
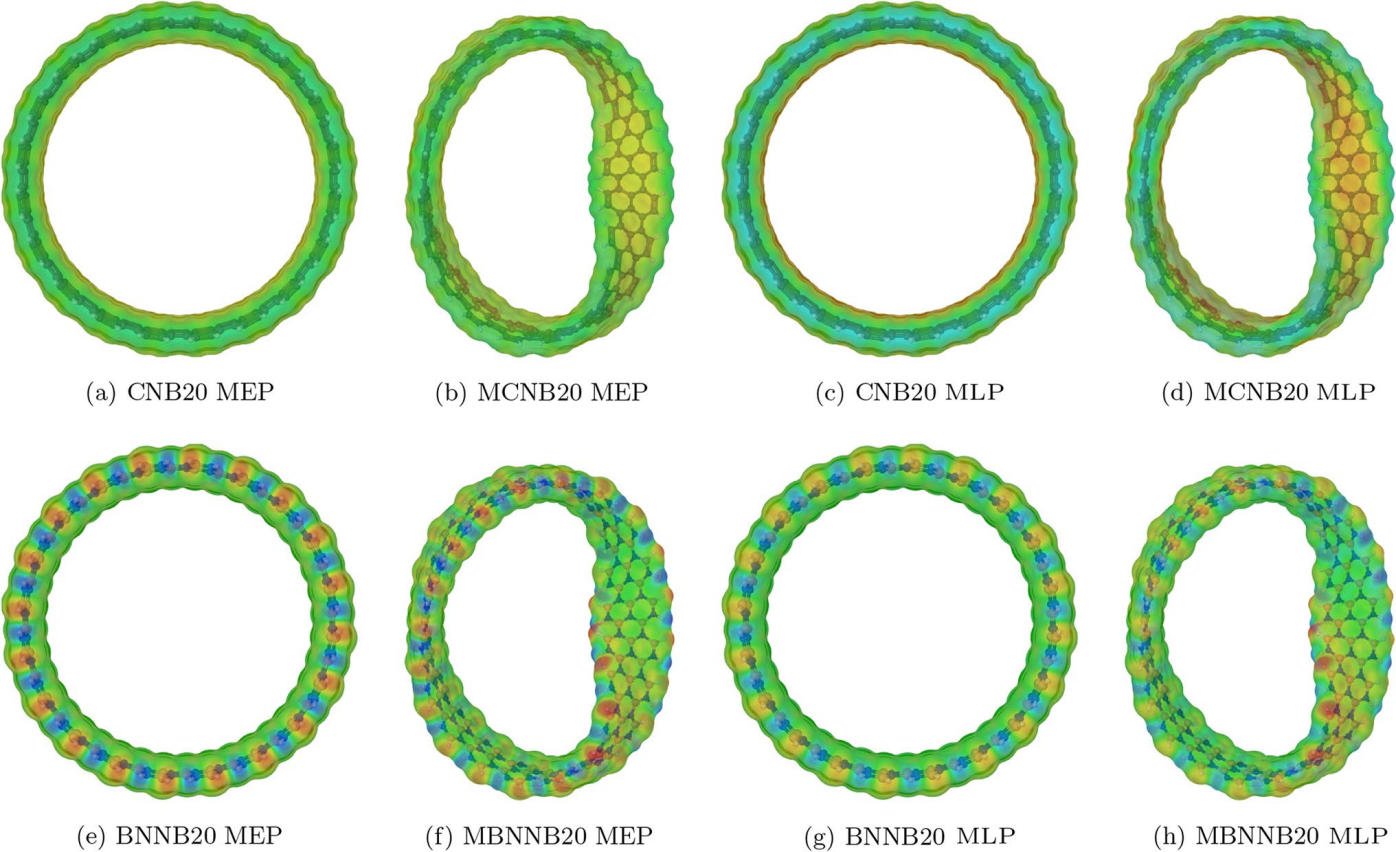
Electrostatic and lipophilic potentials mapped on carbon (top row) and boron-nitride (bottom row) systems surfaces. Red (blue) color represents negative (positive) values. Image rendered with Jmol software [79]

The mapped electrostatic (MEP) and lipophilic (MLP) potentials are shown in Fig. 9. The top (bottom) row presents the data for carbon (boron-nitride) systems with 20 repetitions. In the case of carbon-based nanobelts, both potentials are homogeneously distributed over the whole system’ s surfaces. On the other hand, the Möbius boron-nitride nanobelts show red (negative potential values) and blue (positive potential values) spots in the twist region. The former indicates that those regions are better able to interact with other systems.

By altering the size of the nanobelt, we are able to modify every calculated electronic property, including the molecular hardness, electrophilicity index, HOMO/LUMO values, surface distribution, gap, chemical potential, and electrostatic and lipophilic potentials. In other words, their electronic properties can be manipulated by regulating the growth process of the structures. The consideration of these properties is critical in the logical development of filters, sensors, drug carriers, and other similar devices, as they directly influence the electronic transport, the adsorption and binding processes of small molecules such as clusters of heavy metals, greenhouse gasses, ligands, DNA fragments, and so forth. Based on our prior research examining the interactions between heavy metal clusters of Ni, Cd, and Pb with carbon and boron-nitride nanobelts [80, 81], we discovered that the Möbius nanobelts consistently exhibit superior interaction characteristics (higher binding energy, more affinity, and stronger bonds) than conventional nanobelts. Furthermore, the interacting regions consistently revolve around the torsion.

**Fig. 10:**
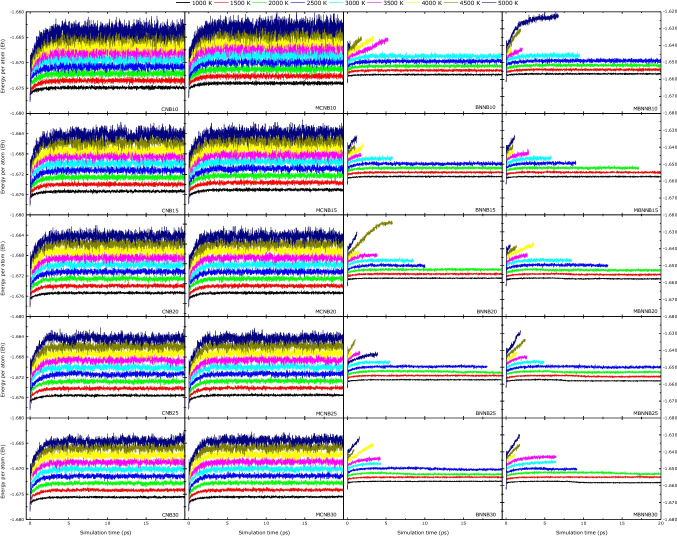
Energy per atom for 20 ps molecular dynamics simulation time

Finally, in order to study the thermal stability of all the systems, molecular dynamics calculations were done for a production time of 20 ps at high temperatures. Figure 10 show the energy per atom as a function of simulation time for all the systems at several temperatures ranging from 1000 K to 5000 K at a 500 K step. The carbon-based nanobelts remain stable at all temperatures during the simulation time, whereas the boron-nitride nanobelts breakout at some temperatures.

**Fig. 11:**
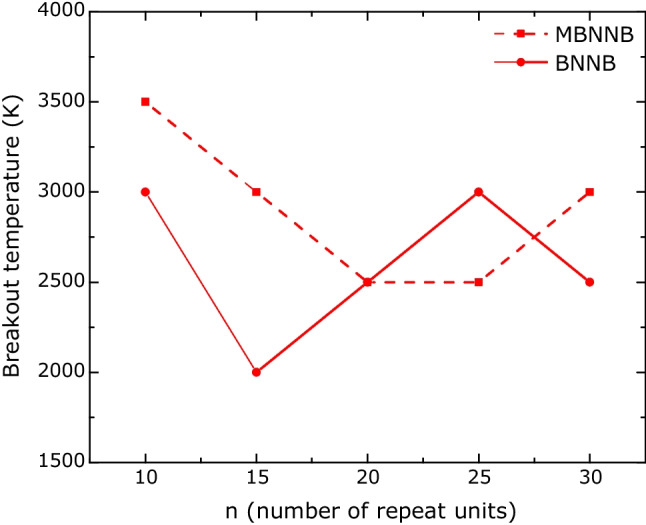
Breakout temperature versus system size

Figure 11 shows the breakout temperature as a function of repetition units. The highest rupture temperature was 3500 K for the smaller Möbius boron-nitride nanobelt (10 repetition units) and the lower breakout temperature was 2000 K for the BNNB15. Three ($n=10,15,30$) of five MBNNB systems show higher stability than BNNB systems; one has the same rupture temperature ($n=20$), and one has a higher breakout temperature ($n=30$).

## Conclusions

In this work, we studied the structural and electronic properties of boron-nitride and carbon-based Möbius nanobelts and compared their properties with those of common nanobelts. For all systems, the main peaks in the infrared calculated spectra are in accordance with the experimental ones, indicating that the theoretical methodology used here is suitable to determine other properties.

The electronic properties show differences between both boron-nitride nanobelts. Whereas the LUMO energy for both systems, BNNB and MBNNB, has similar monotony, the HOMO has shown opposite behavior (different monotony), influencing all the other properties. When compared to BNNBs, MBNNBs have lower values of molecular hardness because the HOMO surface is not spread out in the same way. This unevenness in the HOMO is shown by the fact that the electrostatic and lipophilic potential surfaces had spots in the parts of the HOMO surface with more volume. In the case of carbon-based nanobelts, all the calculated properties show the same behavior for both systems, MCNB and CNB. In contrast to what happened with the boron-nitride system, the HOMO and LUMO surfaces are the same all over the structure of the carbon-based nanobelts. This is reflected in the electrostatic and lipophilic potential surfaces that showed a homogeneous distribution, too. In all cases, the properties vary with the number of repeated units, indicating that it is possible to choose their desired values by changing the size and type of the systems.

The higher temperature molecular dynamics reveal that the carbon-based nanobelts are more stable than the boron-nitride nanobelts, as they did not break apart with the increase in temperature. The rupture temperature for the boron–nitride systems varies with the system size, showing that the MBNNBs have better stability for small sizes than the BNNBs.

### Supplementary Information

**Additional file 1: Fig. S1.** Frontier orbitals (HOMO and LUMO) for all boron-nitride nanobelts. Top to bottom: number of repetitions, *n*, from 10 to 30. **Fig. S2.** Frontier orbitals (HOMO and LUMO) for all Möbius boron-nitride nanobelts. Top to bottom: number of repetitions, *n*, from 10 to 30. **Fig. S3.** Frontier orbitals (HOMO and LUMO) for all carbon nanobelts. Top to bottom: number of repetitions, n, from 10 to 30. **Fig. S4.** Frontier orbitals (HOMO and LUMO) for all Möbius carbon nanobelts. Top to bottom: number of repetitions, *n*, from 10 to 30. **Fig. S5.** Distance distribution (calculations and image produced with OVITO software.

## Data Availability

The raw data required to reproduce these findings is available under request.
