# Supplementary material for: Electronic and structural properties of Möbius boron-nitride and carbon nanobelts
Source: Discov Nano. 2024 Apr 8;19(1):63. doi: 10.1186/s11671-024-03967-0 (PMC11001837; doi:10.1186/s11671-024-03967-0)
Supplement: Supplementary file 1 — Additional file 1: Fig. S1. Frontier orbitals (HOMO and LUMO) for all boron-nitride nanobelts. Top to bottom: number of repetitions, n, from 10 to 30. Fig. S2. Frontier orbitals (HOMO and LUMO) for all Möbius boron-nitride nanobelts. Top to bottom: number of repetitions, n, from 10 to 30. Fig. S3. Frontier orbitals (HOMO and LUMO) for all carbon nanobelts. Top to bottom: number of repetitions, n, from 10 to 30. Fig. S4. Frontier orbitals (HOMO and LUMO) for all Möbius carbon nanobelts. Top to bottom: number of repetitions, n, from 10 to 30. Fig. S5. Distance distribution (calculations and image produced with OVITO software. [file 11671_2024_3967_MOESM1_ESM.pdf]

Supplementary material: Electronic and structural properties of Möbius boron–nitride and carbon nanobelts

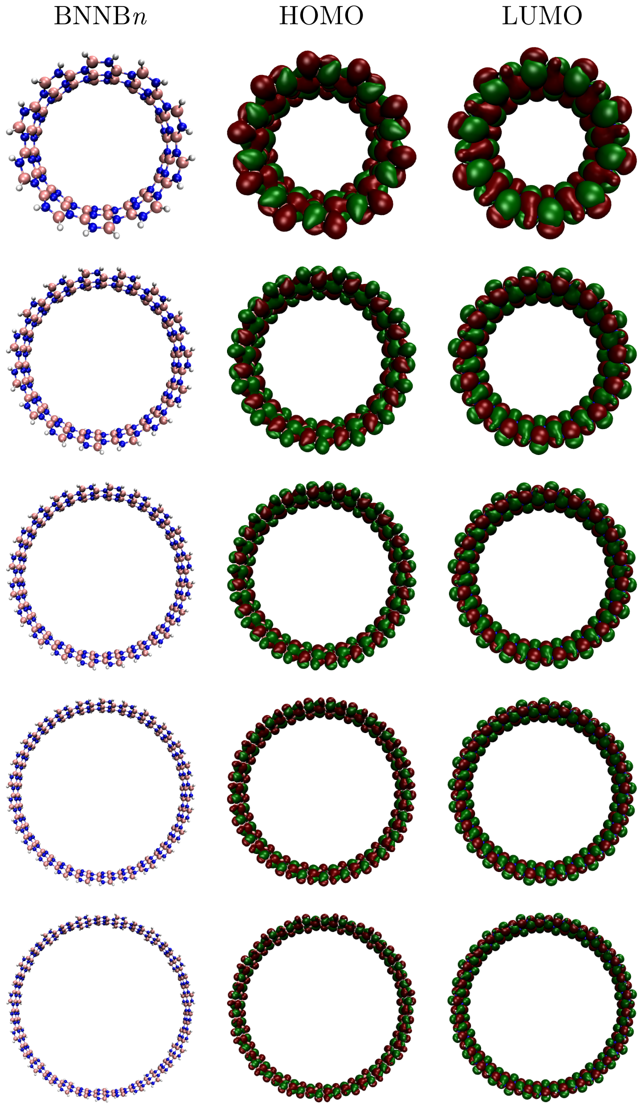

Fig. S1 Frontier orbitals (HOMO and LUMO) for all boron–nitride nanobelts. Top to bottom: number of repetitions, n, from 10 to 30.

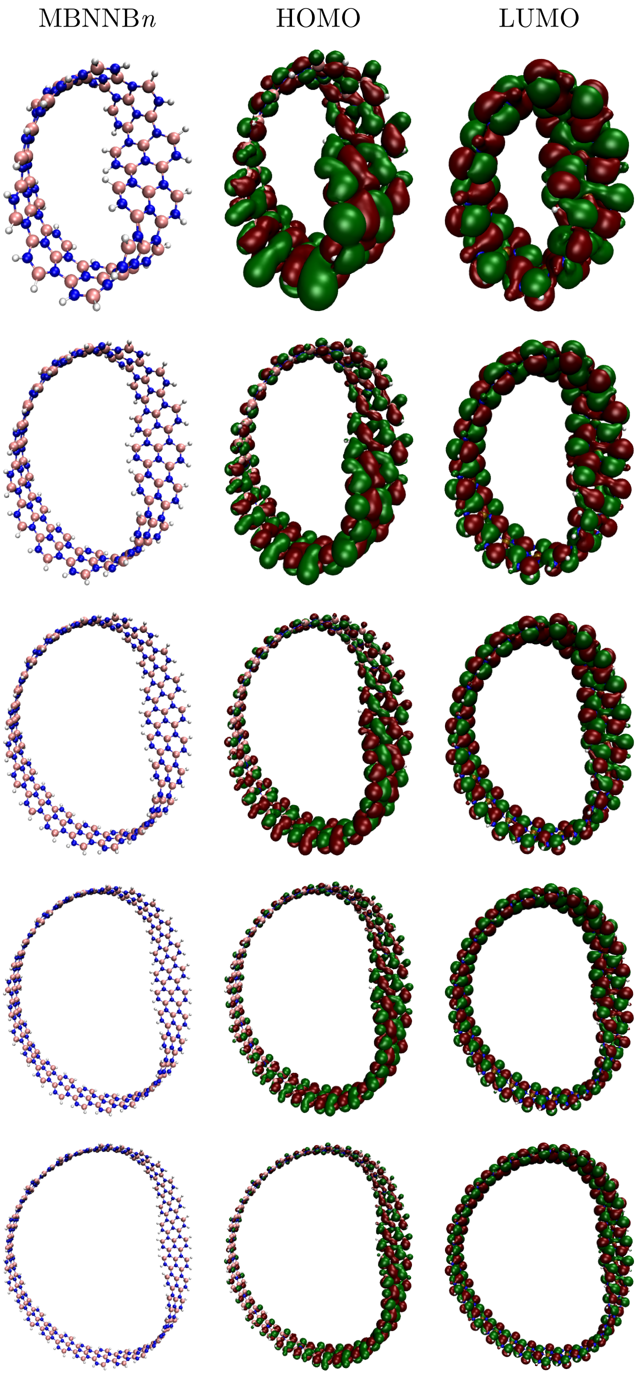

Fig. S2 Frontier orbitals (HOMO and LUMO) for all Möbius boron–nitride nanobelts. Top to bottom: number of repetitions, n, from 10 to 30.

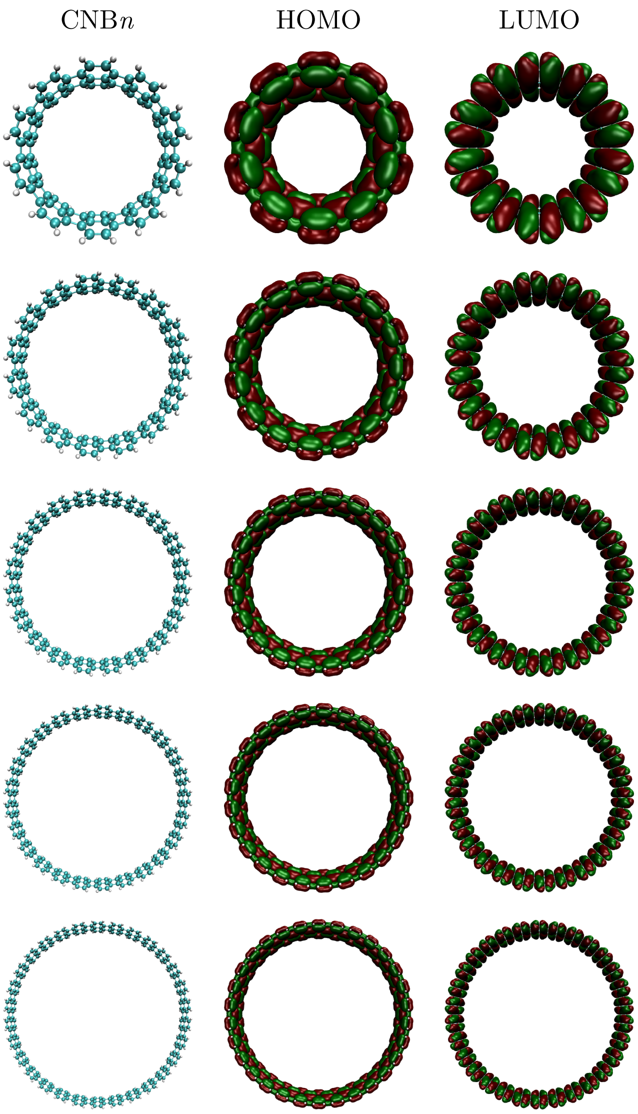

Fig. S3 Frontier orbitals (HOMO and LUMO) for all carbon nanobelts. Top to bottom: number of repetitions, n, from 10 to 30.

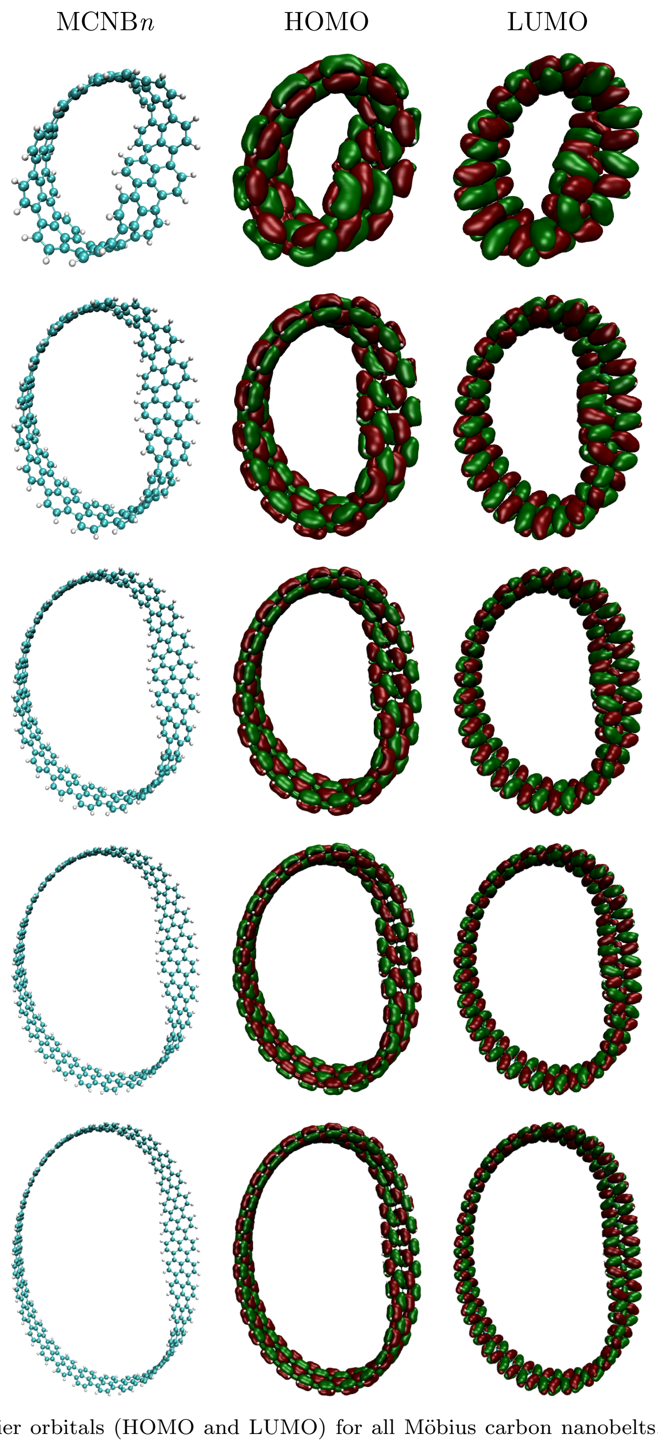

Fig. S4 Frontier orbitals (HOMO and LUMO) for all Möbius carbon nanobelts. Top to bottom: number of repetitions, n, from 10 to 30.

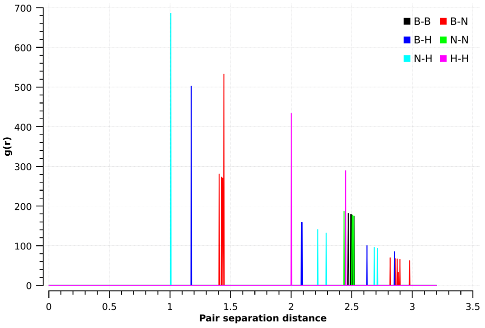

(a) BNNB20x

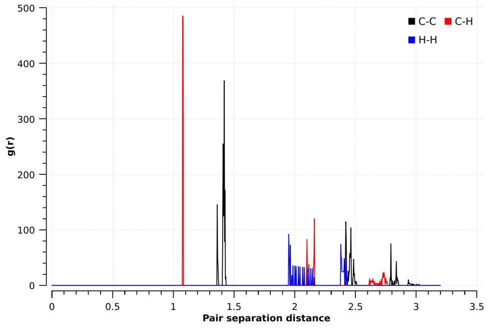

(b) CNB20x

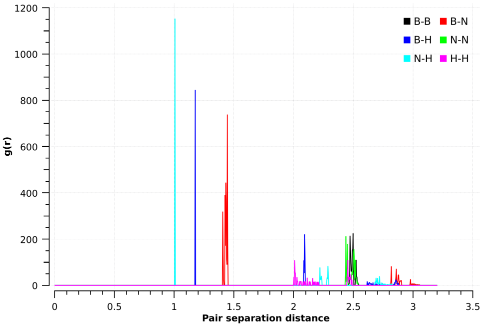

(c) MBNNB20x

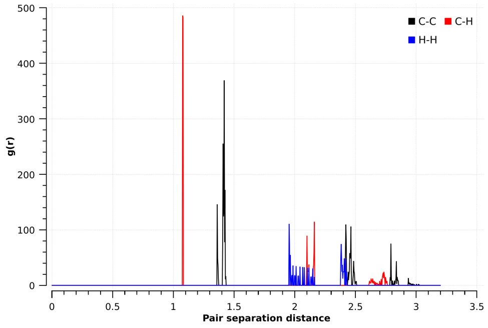

(d) MCNB20x

Fig. S5 Distance distribution (calculations and image produced with OVITO software [S1]).

References

[S1] Stukowski, A.: Visualization and analysis of atomistic simulation data with OVITO–the Open Visualization Tool. Model. Simul. Mater. Sci. Eng. **18**, 015012 (2009) https://doi.org/10.1088/0965-0393/18/1/015012
